# Statins Reduce Hepatocellular Carcinoma Risk in Patients with Chronic Kidney Disease and End-Stage Renal Disease: A 17-Year Longitudinal Study

**DOI:** 10.3390/cancers14030825

**Published:** 2022-02-06

**Authors:** Fung-Chang Sung, Yi-Ting Yeh, Chih-Hsin Muo, Chih-Cheng Hsu, Wen-Chen Tsai, Yueh-Han Hsu

**Affiliations:** 1 Department of Health Services Administration, China Medical University, Taichung 404, Taiwan; fcsung@mail.cmu.edu.tw (F.-C.S.); cch@nhri.edu.tw (C.-C.H.); wtsai@mail.cmu.edu.tw (W.-C.T.); 2 Management Office for Health Data, China Medical University Hospital, Taichung 404, Taiwan; a17776@mail.cmuh.org.tw; 3 Department of Food Nutrition and Health Biotechnology, Asia University, Taichung 413, Taiwan; 4 Division of Family Medicine, Ditmanson Medical Foundation Chia-Yi Christian Hospital, Chia-Yi 600, Taiwan; 07673@cych.org.tw; 5 Graduate Institute of Clinical Medical Science, College of Medicine, China Medical University, Taichung 404, Taiwan; 6 Institute of Population Health Sciences, National Health Research Institutes, Zhunan 350, Taiwan; 7 Department of Family Medicine, Min-Sheng General Hospital, Taoyuan 330, Taiwan; 8 Department of Medical Research, China Medical University Hospital, Taichung 404, Taiwan; 9 Department of Nursing, Min-Hwei Junior College of Health Care Management, Tainan 736, Taiwan; 10 Division of Nephrology, Department of Internal Medicine, Ditmanson Medical Foundation Chia-Yi Christian Hospital, Chia-Yi 600, Taiwan

**Keywords:** chronic kidney failure, hepatocellular carcinoma, hydroxymethylglutaryl-CoA reductase inhibitors, renal dialysis, retrospective cohort study, statins

## Abstract

**Simple Summary:**

Statins are medicines used to treat patients with high lipid levels (hyperlipidemia). Studies have reported that patients undergoing statin therapy are at reduced risk of developing liver cancer. In this study, we compared the risk of developing liver cancer among hyperlipidemic patients with and without statin therapy in three patient groups classified by renal function: normal renal function (NRF) group, chronic kidney disease (CKD) not requiring dialysis, and dialysis-dependent end stage of real disease (ESRD). Our results showed that the risk of developing liver cancer increased progressively from NRF group to CKD and ESRD groups, but was lower for patients receiving statins treatment than non-treated patients. We also found that the statin therapy effectiveness was better in patients taking hydrophilic statins than in those taking lipophilic statins, and in patients taking statin-ezetimibe combination than in those taking statin alone, particularly in the NRF group. Ezetimibe is also an effective option of treating hyperlipidemia.

**Abstract:**

Hepatocellular carcinoma (HCC) is the most common cancer in end-stage renal disease (ESRD) patients in Taiwan. Whether statin therapy associated with the HCC risk in hyperlipidemic patients with chronic kidney disease (CKD) and ESRD is unclear. Using population-based insurance claim data from Taiwan, we identified from hyperlipidemic patients taking statins or not (677,364 versus 867,707) in 1999–2015. Among them, three pairs of propensity score matched statin and non-statin cohorts were established by renal function: 413,867 pairs with normal renal function (NRF), 46,851 pairs with CKD and 6372 pairs with ESRD. Incidence rates of HCC were compared, by the end of 2016, between statin and non-statin cohorts, between hydrophilic statins (HS) and lipophilic statins (LS) users, and between statin-ezetimibe combination therapy (SECT) and statin monotherapy (SM) users. The HCC incidence increased progressively from NRF to CKD and ESRD groups, was lower in the statin cohort than in the non-statin cohort, with the differences of incidence per 10,000 person-years increased from (7.77 vs. 21.4) in NRF group to (15.8 vs. 37.1) in CKD group to (19.1 vs. 47.8) in ESRD group. The incidence increased with age, but the Cox method estimated hazard ratios showed a greater statin effectiveness in older patients. Among statin users, the HCC incidence was lower in HS users than in LS users, and lower in SECT users than in SM users, but the difference was significant only in the NRF group. Hyperlipidemic patients with CKD and ESRD receiving statins are at reduced HCC risks; the treatment effectiveness is superior for HS users than for LS users, and for SECT users than for SM users, but not significant.

## 1. Introduction

The risk of developing cancer is higher in dialysis patients than in the general population, with the excess risk up to 70% [1,2,3]. Among cancers, the risk of hepatocellular carcinoma (HCC) could be 20% to 60% higher in dialysis population than in the general population [1,2,3]. HCC is one of the most common cancers worldwide in the past decades, with over 900,000 incident cases in 2020 [4]. Taiwan has the highest incidence and prevalence of end-stage renal disease (ESRD) in the world [5]. In Taiwan, HCC is the most common form of cancer diagnosed among dialysis patients [6]. Studies have reported that dialysis patients with hepatitis B infection, hepatitis C infection and cirrhosis, non-alcoholic fatty liver disease (NAFLD), and alcoholic liver disease are at an increased risk of developing HCC [7,8,9,10]. In fact, a changing scenario of HCC risk factors emerged in recent years, with increasing proportions of HCC risk related to non-viral metabolic liver disease. An Italian nation-wide study recently reported that the HCC risk might be elevated for up to 37% relating to patients with NAFLD who have higher chance to assume statins [11].

Statins are currently the most widely used group of cholesterol-lowering medications, which may provide clinical benefits in reducing cardiovascular events and increasing long-term survivals [12]. Statins have also been suggested to have pleotropic effects, such as anti-inflammation, anti-oxidant, and anti-proliferative effects [13,14,15,16]. However, Collins et al. stated in a comprehensive review that statin therapy might provide no clinical benefits for cancer patients [17]. More recent research reported anticancer efficacy of statins on several cancers, including ovarian cancer, prostate cancer and HCC [18,19,20]. Systemic review and meta-analyses also reported that statin therapy could reduce over 40% of HCC incidence in the general population [20,21].

It remains unclear whether statin therapy is associated with a reduced risk of HCC in hyperlipidemic patients with CKD and ESRD. In this study, we aimed to fill the evidence gap by conducting a population-based propensity-score matched study, comparing the HCC risk between hyperlipidemic patients with and without statin therapy, using insurance claims data of Taiwan. The statin effectiveness was evaluated for three groups of insured people: individuals who had normal renal function (NRF), chronic kidney disease (CKD) and ESRD. We compared the treatment effectiveness between overall statin and non-statin cohorts, between hydrophilic statins (HS) and lipophilic statins (LS) users, and between statin-ezetimibe combination therapy (SECT) and statin monotherapy (SM) users.

## 2. Materials and Methods

### 2.1. Data Source

We used the Health and Welfare Data Science Center database of all-population, obtained from the Ministry of Health and Welfare of Taiwan, consisting of the National Health Insurance Research database (NHIRD), Registry for catastrophic illness, and death registry. Information available in NHIRD included demographic status of insured population, and outpatient claims and inpatient claims consisting of medical treatments, medications and cost. These data were linked by re-coded identifications to protect the privacy of the insured individuals. Drugs and disease classifications conformed to the Anatomical Therapeutic Chemical (ATC) Classification System and the International Statistical Classification of Diseases and Related Health Problems 9th Revision (ICD-9) for disease before 2016, and ICD-10 since 2016. This study was approved by the Research Ethics Committee at China Medical University and Hospital (CRREC-107-021), and Ditmanson Medical Foundation Chiayi Christian Hospital (CYCH-IRB-2019063).

### 2.2. Study Subjects

**Figure 1:**
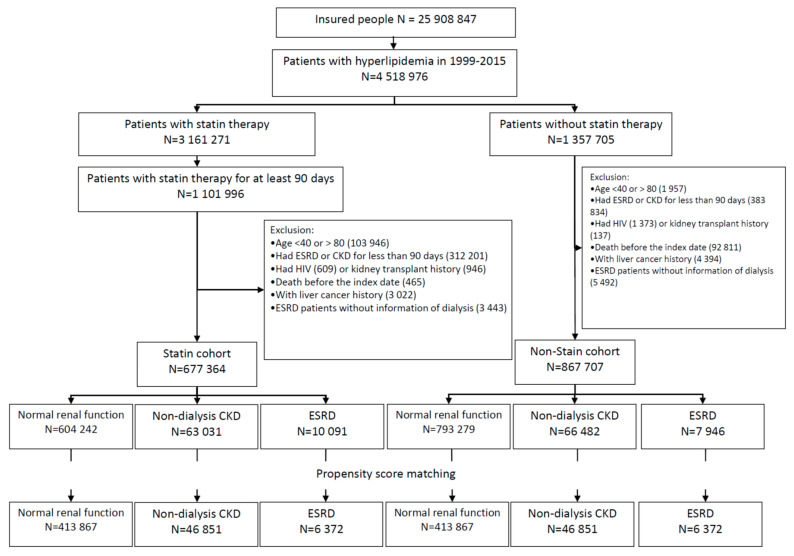
Flow Chart for Identifying Study Cohorts.

We identified 4,518,976 patients with hyperlipidemia (ICD-9: 272/ICD-10: E780-E785) in 1999–2015 from the database. Among the 3,161,271 patients (~70%) receiving statin therapy, 1,101,996 patients had used the medication continuously for at least 90 days (Figure 1). We excluded patients aged <40 or >80 (*n* = 103,946), patients with the history of ESRD or CKD for less than 90 days (*n* = 312,201), HIV (*n* = 609) or kidney transplant (*n* = 946). Patients deceased at the baseline (*n* = 465), patients with liver cancer history (*n* = 3022), and ESRD patients without dialysis information (*n* = 3443) were also excluded. Overall, 677,364 patients were stratified into the three statin sub-cohorts based on the renal function: patients with NRF, with non-dialysis CKD, and with ESRD. The 91st day of statin therapy was designated as the index date.

Applying exclusion criteria similar to the statin cohort, we identified 867,707 patients without statin therapy eligible for the non-statin sub-cohorts. The index date was randomly assigned to each person with the date between the 91st day after hyperlipidemia diagnosis and the date of death. Hyperlipidemic patients without statin therapy were also classified into three subgroups by the renal function as shown in Figure 1. From each subgroup, we further randomly selected a non-statin cohort with the sample size similar to the corresponding statin sub-cohort, frequency matched by propensity score (PS). PS was calculated by multivariable logistic regression, based on renal function status, with information on age, gender, monthly income, living area, comorbidity, and the year with hyperlipidemia diagnosed in the NRF subgroup. In the CKD subgroup, we added the year of CKD diagnosis for adjustment. In the ESRD subgroup, we further added the year of ESRD diagnosis and the type of dialysis for adjustment.

### 2.3. Outcome and Comorbidity

All individuals in the study cohorts were followed from their index dates to the occurrence of HCC (ICD-9/ICD-10: 155/C22), deaths withdrawal from the insurance, or until the end of 2016. The primary outcome was the diagnosis of HCC. Age, gender, income (≤19,200, 19,201–21,900, 21,901–36,300, >36,300 NTDs), living area (north, center, south, eastern, and off islands), comorbidity, and metformin use were considered as covariates. We included diabetes (DM), hypertension, non-alcoholic fatty liver disease (NAFLD), alcohol-related liver diseases (ALD), hepatitis B virus infection (HBV), hepatitis C virus infection (HCV) and cirrhosis as comorbidities that might be associated with the outcomes. All comorbidities were defined within one year before the index date.

### 2.4. Statistical Analysis

Data analysis first compared baseline distributions of demographic status, comorbidities and propensity scores between each pair of statin and non-statin cohorts in the 3 renal groups of NRF, CKD, and ESRD. We calculated standardized difference of each variable between each pair of cohorts. Cumulative incidence of HCC was estimated and plotted for each cohort using cumulative incidence function, and the Gray’s test was used to examine the difference between each pair of cohorts. The incidence was calculated as the number of incident HCC cases divided by the sum of follow-up person-years in each cohort. Incidence rates of HCC were calculated by age (45–54, 55–64, and 65–80 years) for each renal group. Cox proportional hazards regression analysis was used to estimate the statin cohort to non-statin cohort adjusted hazard ratio (aHR) and 95% confidence interval (CI) of HCC by age with three adjustment models: controlling for matched pair in model 1. Considering that clinicians might change or stop statin prescription, we used time-dependent Cox method to estimate the aHR of HCC in model 2. We further assessed the sub-distribution hazard ratio (SHR) of HCC accounting for the competing risk of death in model 3. Due to the limitation of the computer efficiency, we randomly select 1/10 paired-patients in the NRF subgroup to assess aHRs in models 2 and 3. We also calculated hydrophilic statins (HS) users and lipophilic statins (LS) users to non-statin users aHRs, and calculated SECT users and SM users to non-statin users aHRs. Pravastatin and rosuvastatin were types of HS, and simvastatin, lovastatin, fluvastatin, atorvastatin, cerivastatin and pitavastatin were types of LS, available to be prescribed for patients. All statistical tests were two-sided, and the statistical significance was defined as *p*-value < 0.05. All analyses were used SAS, version 9.4 (SAS Institute, Cary, NC, USA).

## 3. Results

We established a statin cohort and a non-statin cohort in each of the three renal-function-based groups: 413,867 in NRF pairs, 46,851 in CKD pairs, and 6372 in ESRD pairs (Figure 1). The CKD cohorts were older with more men than the other two subgroups (Table 1). Distributions of income, living area and comorbidities between the statin cohort and non-statin cohort in each group were similar. The average follow-up periods were 6.28 ± 4.29, 4.95 ± 4.00 and 3.58 ± 3.22 years for NRF, CKD and ESRD groups, respectively.

### 3.1. Cumulative HCC Incidence

**Figure 2:**
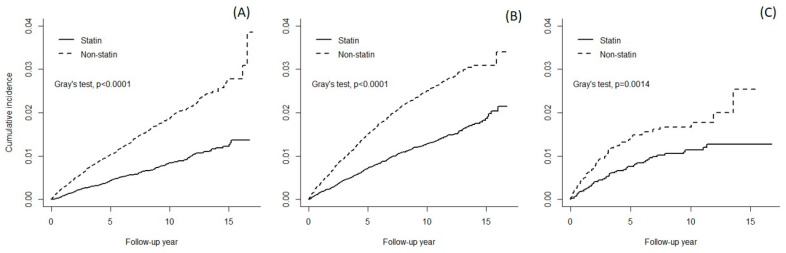
Cumulative incidence function of hepato-cellular carcinoma among three study groups. (**A**) Normal Renal Function, (**B**) Chronic Kidney Disease without dialysis, (**C**) End Stage Renal Disease.

After 17 follow-up years, the cumulative incidence in statin users was about 2.70% lower than that in non-statin users (1.18% vs. 3.88%, Gray’s test *p* < 0.0001) in the NRF group (Figure 2). The cumulative incidence gap between the statin cohort and non- statin cohort decreased to 1.21% (2.01% vs. 3.22%, Gray’s test *p* < 0.0001) in the CKD group and to 1.31% (1.17% vs. 2.48%, Gray’s test *p* = 0.0014) in the ESRD group.

### 3.2. HCC Incidence and Statin Cohort to Non-Statin Cohort HRs of HCC

The overall HCC incidence in the NRF group was 13.5 per 10,000 person-years (or 2.8-fold) greater in the non-statin cohort than in the statin cohort (21.4 versus 7.77 per 10,000 person-years, or 5134 versus 2180 cases) (Table 2). The differences of HCC incidence rates between the non- statin cohort and the statin cohort increased to 21.3 per 10,000 person-years (37.1 versus 15.8 per 10,000 person-years) in the CKD group and to 28.7 (47.8 versus 19.1 per 10,000 person-years) in the ESRD group. The corresponding statin cohort to non- statin cohort aHRs estimated in model 1 were 0.36 (95% CI = 0.35–0.38) in the NRF group, 0.42 (95% CI = 0.38–0.48) in the CKD group and 0.41 (95% CI = 0.29–0.59 in the ESRD group. The incidence increased with age in all cohorts. However, the estimated statin cohort to the non-statin cohort HRs were lower in older statin users in all three renal groups.

### 3.3. Effectiveness in LS and HS Users and in SM and SECT Users

Table 3 shows that the HCC incidence rate was lower in HS users than in LS users in all three renal groups. The aHR of developing HCC was 0.28 (95% CI = 0.26–0.31) for HS users, compared to non-statin users, in the NRF group. The corresponding aHRs were 0.36 (95% CI = 0.30–0.44) in the CKD group and 0.38 (95% CI = 0.21–0.70) in the ESRD group. However, the superior effectiveness of HS therapy, compared to LS therapy, was significant for the NRF group only. Table 3 also shows that the HCC incidence was lower in the SECT users than SM users in all 3 renal groups, but the SECT users to SM users aHR of HCC was significant for the NRF group only.

## 4. Discussion

In this study, we noted a progressively increased risk of HCC from the NRF cohort to the CKD and ESRD cohorts. We also observed a reduced HR of developing HCC for over 50% in hyperlipidemic patients with NRF, CKD or ESRD receiving statin therapy, based on the models adjusted for matched pair and the time-dependent Cox regression model. The age-specific aHR of HCC declined in statin users with increasing age, indicating the statin treatment efficacy is superior in the elderly groups. Among statin users, the incidence rates of HCC were lower in the HS users than the LS users, although the reduction levels between the two types of medication seemed not significant for patients with CKD and ESRD, maybe due to the small outcome numbers. Our evaluation also showed that patients receiving SECT had the incident HCC reduced further than patients receiving SM, but also was significant only in the NRF group.

Our findings not only add information on patients with CKD and ESRD but also extend previous meta-analyses findings linking statin therapy to a reduced risk of HCC [20,21,22], in patients with diabetes, cirrhosis or antiviral therapy [20] and patients with non-alcoholic steatohepatitis (NASH) [23]. An earlier case-control research reported that the prolonged use of statins was associated with increased risk of colorectal cancer, bladder cancer and lung cancer [24]. An earlier review study also failed to find clinical benefits of statin therapy for cancer patients [17]. However, prolific clinical studies and basic laboratory studies have reported evidence supporting the anti-cancer effects of statins [13,18,20,23,25].

Known as HMG-CoA reductase inhibitors, statins inhibit the rate-limiting enzyme in the mevalonate pathway; while many cancer cells depend on the mevalonate pathway for growth and survival [26]. Studies have reported that statins can function as a blockade of diverse carcinogenic pathways [27]. A low dose of pitavastatin can inhibit NF-kappaB activation to reduce the TNF-alpha induced IL-6. Statins can regulate the Rho-dependent kinase pathway, reducing the cancer risk. Pathways regulated by Myc, PI3KAkt, integrins or Hippo signaling can also exert the role of chemoprotective effect on cancers in statin users, including on HCC. However, little is known about the relative impact of statin on HCC chemoprevention in CKD and ESRD groups.

A previous study has reported that aspirin, a low-cost medication for cardiovascular protection, was associated with reduced incidence of and lower mortality from HCC in patients with viral hepatitis infection [28]. Ielasi et al. also reported recently that aspirin use could improve survival in HCC patients who received sorafenib treatment [29]. Statins could also benefit patients with reduced risk of recurrence and improved survival after a surgical resection or liver transplant [30]. Since patients with hypertension were included in our study, for whom aspirin and statin were frequently prescribed simultaneously, the effect of aspirin might exist in research addressing the efficacies of statins.

The statin therapy effectiveness may vary by ethnic group, disease type and age. Our findings demonstrated a trend of lower adjusted HR of HCC in HS users than in LS users. This finding was not in line with the meta-analysis of 18 studies involving 1,611,596 patients, which suggested that the associated reduction in risks of HCC were similar in HS users and in LS users [21]. Also, the result was not in accord with a Swedish population-based cohort study, which reported a significantly reduced HCC risk among LS users with viral hepatitis, but not among HS users [31]. Ethnical characteristics (Taiwanese versus Swedish) might account for the discrepancy. For instance, plasma exposure to rosuvastatin (hydrophilic class) and its metabolites was observed significantly higher in Asians than in Caucasians [32]. Two polymorphisms in the organic anion-transporting polypeptide 1B1 (OATP1B1; encoded by SLCO1B1) and the intestinal breast cancer resistance protein (BCRP; encoded by ABCG2) genes may contribute to the ethnicity-dependent variability in the pharmacogenetics of rosuvastatin [33].

Our results showed a progressively lower hazards of incident HCC with increasing age in patients with CKD and ESRD. Few previous studies have reported differential efficacies of statin drugs on increasing age. A meta-analysis by Cholesterol Treatment Trialists’ Collaboration reported statins reduced risk of vascular events irrespective of ages, with less benefit for patients older than 75 years [34]. A retrospective cohort study in Spain with 46,864 participants aged 75 and older evaluated mortality risk from atherosclerotic cardiovascular disease associated with statin for patients with and without diabetes. The effectiveness was significant for diabetic patients aged 75–84, but not older. The benefit of statin therapy was not significant for patients without diabetes [35]. Further research is needed to evaluate the association of age-related efficacies of statin on the HCC risk in patients with renal dysfunction.

Our study also showed that patients receiving the SECT had a lower HCC incidence than patients with SM in all three groups. Although, a significant reduction was observed only in the NRF group. Smaller sizes of patients and small numbers of events in CKD and ESRD groups might account for the lack of significance level. However, the results hint of an effectiveness trend of SECT in these patients and deserve our attention. Since the HCC incidence in SECT users in the ESRD group was lower than that in the general statin cohort (14.4 versus 19.1 per 10,000 person-years), the effectiveness of SECT seems worthwhile to care ESRD. The component of ezetimibe in SECT may play an important role. A meta-analysis has reported the benefits of ezetimibe in reducing deaths from all-cause and cardiovascular disease but not from new cancer [36]. Another systemic review and meta-analysis also reported that trials of Ezetimibe ± simvastatin failed to demonstrate effectiveness in reducing deaths from various diseases [37]. An animal study showed ezetimibe suppressed the development of liver tumors by inhibiting angiogenesis in mice with hypercholesterolemia [38], which was consistent with our findings. Few previous clinical studies have investigated the HCC risk in patients with renal dysfunction.

The major strength of this study is that the large population-based longitudinal data allowed us to conduct a long follow-up study, controlling for multiple confounding factors, including traditional HCC risk factors and metformin use. We also made great efforts to reduce bias and confounding effects, by addressing the immortal time bias via study design, establishing PS matched cohorts, conducting time-variable model and competing risk model against death. The results of sensitivity analyses were agreeable to the main results. However, in order to evaluate the role of comorbidities on the development of HCC, we further conducted nested case-control analyses, separately, for statin users and non-users. Results show that HBV, HCV and cirrhosis were significant factors associated with developing HCC in both statin user cohort (Table S1) and non-user cohort (Table S2). The risk of HCC associated with these liver disorders were greater for statin non-users than for statin users. Among population with HCC developed, 63.6% (1692/2659) and 70.7% (4197/5937) of cases were not associated with liver disorders in statin users and non-users, respectively. With these efforts, the credibility of our results is enhanced.

We acknowledge several limitations in this study. First, the study was conducted on a health insurance claims database lacking information on certain risk factors of HCC, such as body mass index, aflatoxins exposure, drinking and smoking. We were unable to control these potential confounding factors. However, we included alcohol-related liver disease to adjust for the influence of alcohol intake. Second, we had no access to lab data and details of medical treatment for patients with hyperlipidemia. However, we conducted data analysis using time-dependent model to evaluate the HR associated with the duration of taking statins. Results showed persistent effectiveness trends. Further investigations on the relationships between effectiveness and dosages of statins may be considered to assist clinical novelty in real-world practices. Finally, the main underlying factors of HCC in Taiwan are different from those in the Western countries. Generalizability of our findings to other healthcare systems may not be feasible.

## 5. Conclusions

In conclusion, hyperlipidemic patients with NRF, CKD or ESRD receiving statins were at a reduced risk of developing HCC. The overall HCC incidence in statin users was lowered for 63.7%, 57.4 % and 60.0%, respectively, in the three groups of patients. The risk reductions seemed stronger with increasing age. Patients receiving HS tended to have a lower HCC risk than those receiving LS have; likewise, patients receiving SECT seemed to have lower risk than those receiving SM. However, it could be worthwhile to use SECT to care ESRD in papulation with a high prevalence of the renal disorder. Further prospective research will be needed to confirm our findings.

## Figures and Tables

**Table 1:** Distribution of age, sex and comorbidity compared between propensity score matched statin and non-statin cohorts in three study groups.

Variable	Normal Renal Function					CKD without Dialysis					ESRD				
	Non-Statin *N* = 413,867		Statin *N* = 413,867		Standardized Difference	Non-Statin *N* = 46,851		Statin *N* = 46,851		Standardized Difference	Non-Statin *N* = 6372		Statin *N* = 6372		Standardized Difference
**Age, mean (SD)**	58.8	(10.6)	58.9	(9.84)	0.009	64.9	(10.7)	64.9	(9.93)	0.001	63.5	(10.3)	63.1	(9.64)	0.037
**Men, *n* (%)**	207,043	50.0	504,001	49.3	0.015	26,664	56.9	26,289	56.1	0.016	3244	50.9	3161	49.6	0.026
**Income, NTD**															
<19,200	88,484	21.4	88,978	21.5	0.003	11,699	25.0	11,677	24.9	0.001	1730	27.2	1737	27.3	0.002
19,200–21,900	128,311	31.0	128,689	31.1	0.002	14,859	31.7	15,031	32.1	0.008	2244	35.2	2253	35.4	0.003
21,901–36,300	78,194	18.9	77,457	18.7	0.005	9242	19.7	9099	19.4	0.008	1125	17.7	1109	17.4	0.007
>36,300	118,878	28.8	118,743	28.7	0.005	11,051	23.6	11,044	23.6	0.008	1273	20.0	1273	20.0	0.007
**Living area**															
North	191,518	46.3	192,042	46.4	0.003	18,104	38.6	17,885	38.2	0.010	2473	38.8	2502	39.3	0.009
Central	79,214	19.1	78,772	19.0	0.003	9811	20.9	9905	21.1	0.005	1320	20.7	1317	20.7	0.001
South	119,741	28.9	119,169	28.8	0.003	16,312	34.8	16,369	34.9	0.003	2216	34.8	2204	34.6	0.004
East and Offshore Islands	23,394	5.65	23,884	5.77	0.003	2624	5.60	2692	5.75	0.003	363	5.70	349	5.48	0.004
**Comorbidity, *n* (%)**															
DM	135,188	32.7	145,069	35.1	0.050	20,619	44.0	21,435	45.8	0.035	3597	56.5	3609	56.6	0.004
Hypertension	233,360	56.4	231,858	56.0	0.007	35,756	76.3	36,001	76.8	0.012	4689	73.6	4664	73.2	0.009
NAFLD	7821	1.89	7859	1.90	0.001	806	1.72	829	1.77	0.004	26	0.41	33	0.52	0.016
ALD	2520	0.61	2387	0.58	0.004	289	0.62	288	0.61	0.000	10	0.16	10	0.16	0.000
HBV	12,265	2.96	12,146	2.93	0.002	1283	2.74	1263	2.70	0.003	266	3.55	202	3.17	0.021
HCV	4735	1.14	4512	1.09	0.005	862	1.84	803	1.71	0.010	260	4.08	237	3.72	0.019
Cirrhosis	3214	0.78	2810	0.68	0.011	726	1.55	624	1.33	0.018	140	2.20	127	1.99	0.014
**Metformin use**	86,387	20.9	98,503	23.8	0.070	11,944	25.5	12,974	27.7	0.050	212	3.33	2008	3.26	0.004
**Hemodialysis**											5843	91.7	5788	90.8	0.031

SD, standard deviation; NTD, New Taiwan Dollar; DM, diabetes mellitus; NAFLD, non-alcoholic fatty liver disease; ALD, alcohol-related liver diseases; HBV, hepatitis B virus; HCV, hepatitis C virus.

**Table 2:** Incidence and statin cohort to non-statin cohort hazard ratio of hepatocellular carcinoma in three study groups by age.

	Non-Statin			Statin			Statin to. Non-Statin Hazard Ratio (95% Confidence Interval)		
Outcome	Event *n*	PYs	Rate, per 10,000 PYs	Event *n*	PYs	Rate, per 10,000 PYs	Model 1	Model 2	Model 3
**NRF**	5134	2,394,676	21.4	2180	2,807,446	7.77	0.36 (0.35–0.38) ***	0.29 (0.22–0.39) ***	0.46 (0.39–0.53) ***
40–54 years	1164	1,047,495	11.1	555	1,094,486	5.07	0.45 (0.41–0.50) ***	0.33 (0.19–0.59) ***	0.39 (0.29–0.54) ***
55–64	1624	711,419	22.8	704	927,857	7.59	0.33 (0.30–0.36) ***	0.28 (0.17–0.45) ***	0.52 (0.40–0.67) ***
65–80	2346	635,763	36.9	921	785,103	11.7	0.32 (0.30–0.35) ***	0.28 (0.18–0.42) ***	0.43 (0.34–0.54) ***
**CKD**	720	194,009	37.1	425	269,655	15.8	0.42 (0.38–0.48) ***	0.50 (0.42–0.60) ***	0.55 (0.49–0.62) ***
40–54	65	46,151	14.1	47	53,622	8.77	0.58 (0.40–0.85) **	0.85 (0.52–1.37)	0.61 (0.42–0.90) *
55–64	162	50,157	32.3	98	76,094	12.9	0.39 (0.30–0.50) ***	0.55 (0.39–0.77) ***	0.46 (0.36–0.60) ***
65–80	493	97,701	50.5	280	139,938	20.0	0.40 (0.34–0.46) ***	0.43 (0.34–0.54) ***	0.56 (0.49–0.65) ***
**ESRD**	83	17,356	47.8	54	28,240	19.1	0.41 (0.29–0.59) ***	0.46 (0.27–0.77) **	0.63 (0.45–0.88) **
40–54	14	4855	28.8	12	7695	15.6	0.58 (0.27–1.27)	0.64 (0.22–1.89)	0.85 (0.39–1.85)
55–64	24	5122	46.9	20	9391	21.3	0.45 (0.25–0.81) **	0.43 (0.18–1.03)	0.65 (0.36–1.15)
65–80	45	7379	61.0	22	11,154	19.7	0.33 (0.19–0.56) ***	0.39 (0.18–0.86) *	0.54 (0.32–0.91) *

PYs: person-years; NRF, normal renal function; CKD, non-dialysis chronic kidney disease; ESRD, end stage renal disease. Model 1: Adjusted for matched pair. Model 2: Adjusted for age, sex, area, income, comorbidity, and metformin use by time-dependent Cox model. Model 3: Adjusted for age, sex, area, income, comorbidity, and metformin use by competing Cox model (death as competing factor). For ESRD group: hemodialysis was also included in model 2 and SHR measure. * *p* < 0.05, ** *p* < 0.01, *** *p* < 0.001.

**Table 3:** Incidence and statin cohort to non-statin cohort adjusted hazard ratio of hepatocellular carcinoma in three study groups by medication type.

Medication Type	*N*	Event *n*	Rate, per 10,000 PYs	aHR (95% CI) †	*p*	aHR (95% CI) ††	*p*
**NRF**							
Non-statin	413,867	5134	21.4	Ref.			
LS	277,943	1617	8.60	0.40 (0.38–0.43)	<0.0001	Ref.	
HS	135,924	563	6.07	0.28 (0.26–0.31)	<0.0001	0.71 (0.64–0.78)	<0.0001
**CKD**							
Non-statin	46,851	720	37.1	Ref.			
LS	32,526	313	16.7	0.45 (0.39–0.52)	<0.0001	Ref.	
HS	14,325	112	13.5	0.36 (0.30–0.44)	<0.0001	0.81 (0.65–1.00)	0.053
**ESRD**							
Non-statin	6372	83	47.8	Ref.			
LS	4851	42	19.6	0.42 (0.29–0.62)	<0.0001	Ref.	
HS	1521	12	17.7	0.38 (0.21–0.70)	0.002	0.91 (0.48–1.72)	0.760
**NRF**							
Non-statin	413,867	5134	21.4	Ref.			
SM	377,351	2006	7.88	0.37 (0.35–0.39)	<0.0001	Ref.	
SECT	36,516	174	6.66	0.31 (0.27–0.36)	<0.0001	0.85 (0.73–0.99)	0.034
**CKD**							
Non-statin	46,851	720	37.1	Ref.			
SM	42,182	388	16.1	0.43 (0.38–0.49)	<0.0001	Ref.	
SECT	4669	37	13.2	0.35 (0.25–0.49)	<0.0001	0.82 (0.59–1.15)	0.246
**ESRD**							
Non-statin	6372	83	47.8	Ref.			
SM	5910	51	19.5	0.42 (0.29–0.60)	<0.0001	Ref.	
SECT	462	3	14.4	0.31 (0.10–0.99)	0.049	0.75 (0.23–2.39)	0.745

PYs: person-years; NRF, normal renal function; CKD, non-dialysis chronic kidney disease; ESRD, end stage renal disease, †: Hazrd ratio adjusted for matched pair ††: Hazard ratio adjusted for age, sex, area, income, comorbidity, and metformin use. Definition of medication in use: the last time used statin. LS, Lipophic Statin; HS, Hydrophic Statin, SM, Statin monotherapy; SECT, Statin-ezetimibe combination therapy. aHR, adjusted hazard ratio; CI, confidence interval.

## Data Availability

The data presented in this study are available on request from the corresponding author.

## Supplementary Materials

The following supporting information can be downloaded at: https://www.mdpi.com/article/10.3390/cancers14030825/s1, Table S1: Case-control analysis of hepatocellular carcinoma in statin user cohort; Table S2: Case-control analysis of hepatocellular carcinoma in the non-Statin user cohort.
