# Supplementary material for: Statins Reduce Hepatocellular Carcinoma Risk in Patients with Chronic Kidney Disease and End-Stage Renal Disease: A 17-Year Longitudinal Study
Source: Cancers (Basel). 2022 Feb 6;14(3):825. doi: 10.3390/cancers14030825 (PMC8834435; doi:10.3390/cancers14030825)
Supplement: Supplementary file 1 [file cancers-14-00825-s001.zip › cancers-1572217-supplementary.pdf]

cancers

Supplementary Materials

Statins reduce hepatocellular carcinoma risk in patients with chronic kidney disease and end-stage renal disease, a 17-year longitudinal study

Fung-Chang Sung, Yi-Ting Yeh, Chih-Hsin Muo, Chih-Cheng Hsu, Wen-Chen Tsai and Yueh-Han Hsu

Table S1. Case-control analysis of hepatocellular carcinoma in statin user cohort

	HCC N=2659		Non-HCC N=464431		Total N=467090		Odds ratio (95% confidence interval)			
Variable	n	%	n	%	n	%	Crude	p	Adjusted	p
Age, year										
40-54	614	23.1	157512	33.9	158126	33.9	Ref.		Ref.	
55-64	822	30.9	156756	33.8	157578	33.7	1.35 (1.21-1.49)	<0.0001	1.55 (1.40-1.73)	<0.0001
65-80	1223	46.0	150163	32.3	151386	32.4	2.09 (1.90-2.30)	<0.0001	2.50 (2.25-2.77)	<0.0001
Sex										
Men	1837	69.1	231614	49.9	233451	50.0	2.25 (2.07-2.44)	<0.0001	2.35 (2.16-2.56)	<0.0001
Women	822	30.9	232817	50.1	233639	50.0	Ref.			
Income, NTD										
<16500	515	19.4	101877	21.9	102392	21.9	1.30 (1.13-1.49)	0.0002	1.23 (1.07-1.42)	0.003
16500-21900	1181	44.4	144792	31.2	145973	31.3	2.10 (1.86-2.36)	<0.0001	2.09 (1.85-2.36)	<0.0001
21901-31800	340	12.8	87325	18.8	87665	18.8	Ref.		Ref.	
>31800	623	23.4	130437	28.1	131060	28.1	1.23 (1.08-1.40)	0.003	1.34 (1.17-1.54)	<0.0001
Living area										
North	987	37.1	211442	45.5	212429	45.5	Ref.		Ref.	
Central	467	17.6	89527	19.3	89994	19.3	1.12 (1.00-1.25)	0.049	0.95 (0.84-1.06)	0.326
South	1007	37.9	136735	29.4	137742	29.5	1.58 (1.45-1.72)	<0.0001	1.26 (1.15-1.38)	<0.0001
East and Offshore Islands	198	7.45	26727	5.75	26925	5.76	1.59 (1.36-1.85)	<0.0001	1.22 (1.04-1.43)	0.013
Comorbidity										
DM										
No	1269	47.7	295708	63.7	296977	63.6	Ref.		Ref.	

Yes	1390	52.3	168723	36.3	170113	36.4	1.92 (1.78-2.07)	<0.0001	1.63 (1.47-1.81)	<0.0001
Hypertension										
No	1013	38.1	193554	41.7	194567	41.7	Ref.		Ref.	
Yes	1646	61.9	270877	58.3	272523	58.3	1.16 (1.07-1.26)	0.0002	1.04 (0.96-1.13)	0.328
NAFLD										
No	2581	97.1	455788	98.1	458369	98.1	Ref.		Ref.	
Yes	78	2.93	8643	1.86	8721	1.87	1.59 (1.27-2.00)	<0.0001	1.38 (1.09-1.74)	0.007
ALD										
No	2609	98.1	461796	99.4	464405	99.4	Ref.		Ref.	
Yes	50	1.88	2635	0.57	2685	0.57	3.36 (2.53-4.46)	<0.0001	1.56 (1.15-2.12)	0.005
HBV										
No	2272	85.5	451207	97.2	453479	97.1	Ref.		Ref.	
Yes	387	14.6	13224	2.85	13611	2.91	5.81 (5.21-6.48)	<0.0001	3.89 (3.45-4.39)	<0.0001
HCV										
No	2414	90.8	459124	98.9	461538	98.8	Ref.		Ref.	
Yes	245	9.21	5307	1.14	5552	1.19	8.78 (7.68-10.0)	<0.0001	4.42 (3.81-5.13)	<0.0001
Cirrhosis										
No	2324	87.4	461205	99.3	463529	99.2	Ref.		Ref.	
Yes	335	12.6	3226	0.69	3561	0.76	20.6 (18.3-23.2)	<0.0001	9.90 (8.66-11.3)	<0.0001
Metformin use										
No	1756	66.0	353649	76.2	355405	76.1	Ref.		Ref.	
Yes	903	34.0	110782	23.9	111685	23.9	1.64 (1.52-1.78)	<0.0001	1.06 (0.95-1.18)	0.299

HCC, hepatocellular carcinoma; DM, diabetes mellitus NTD, New Taiwan Dollar; NAFLD, non-alcoholic fatty liver disease; ALD, alcohol-related liver diseases; HBV, hepatitis B virus; HCV, hepatitis C virus.

Table S2. Case-control analysis of hepatocellular carcinoma in the non-Statin user cohort

	HCC N=5937		Non-HCC N=461153		Total N=467090		OR (95% CI)			
Variable	N	%	n	%	n	%	Crude	p	Adjusted	p
Age, year										
40-54	1243	20.9	167588	36.3	168831	36.2	Ref.		Ref.	
55-64	1810	30.5	139338	30.2	141148	30.2	1.75 (1.63-1.88)	<0.0001	1.84 (1.70-1.98)	<0.0001
65-80	2884	48.6	154227	33.4	157111	33.6	2.52 (2.36-2.70)	<0.0001	2.53 (2.36-2.72)	<0.0001
Sex										
Men	3818	64.3	233133	50.5	236951	50.7	1.76 (1.67-1.86)	<0.0001	1.86 (1.76-1.97)	<0.0001
Women	2119	35.7	228020	49.5	230139	49.3	Ref.		Ref.	
Income, NTD										
<16500	1155	19.5	100758	21.9	101913	21.8	1.24 (1.29-1.56)	<0.0001	1.23 (1.12-1.35)	<0.0001
16500-21900	2830	47.7	142584	30.9	145414	31.1	2.46 (2.26-2.67)	<0.0001	2.20 (2.02-2.40)	<0.0001
21901-31800	709	11.9	87852	19.1	88561	19.0	Ref.		Ref.	
>31800	1243	20.9	129959	28.2	131202	28.1	1.19 (1.08-1.30)	0.0003	1.28 (1.17-1.41)	<0.0001
Living area										
North	2066	34.8	210029	45.5	212095	45.4	Ref.		Ref.	
Central	1108	18.7	89237	19.4	90345	19.3	1.26 (1.17-1.36)	<0.0001	1.05 (0.97-1.13)	0.228
South	2410	40.6	135859	29.5	138269	29.6	1.80 (1.70-1.91)	<0.0001	1.41 (1.33-1.50)	<0.0001
East and Offshore Islands	353	5.95	26028	5.64	26381	5.65	1.38 (1.23-1.55)	<0.0001	1.12 (0.99-1.26)	0.053
Comorbidity										
DM										
No	2978	50.2	304708	66.1	307686	65.9	Ref.		Ref.	
Yes	2959	49.8	156445	33.9	159404	34.1	1.94 (1.84-2.04)	<0.0001	1.45 (1.35-1.56)	<0.0001
Hypertension										
No	2362	39.8	190923	41.4	193285	41.4	Ref.		Ref.	
Yes	3575	60.2	270230	58.6	273805	58.6	1.07 (1.02-1.13)	0.012	0.86 (0.82-0.91)	<0.0001
NAFLD										
No	5802	97.7	452635	98.2	458437	98.2	Ref.		Ref.	
Yes	135	2.27	8518	1.85	8653	1.85	1.24 (1.40-1.47)	0.016	1.07 (0.90-1.28)	0.450
ALD										
No	5833	98.3	458438	99.4	464271	99.4	Ref.		Ref.	

Yes	104	1.75	2715	0.59	2819	0.60	3.01 (2.47-3.67)	<0.0001	1.43 (1.15-1.78)	0.001
HBV										
No	5395	90.9	447921	97.1	453316	97.1	Ref.		Ref.	
Yes	542	9.13	13232	2.87	13774	2.95	3.40 (3.11-3.72)	<0.0001	2.84 (2.58-3.13)	<0.0001
HCV										
No	5338	89.9	455895	98.9	461233	98.8	Ref.		Ref.	
Yes	599	10.1	5258	1.14	5857	1.25	9.73 (8.90-10.6)	<0.0001	5.93 (5.38-6.53)	<0.0001
Cirrhosis										
No	5338	89.9	457672	99.3	463010	99.1	Ref.		Ref.	
Yes	599	10.1	3481	0.75	4080	0.87	14.8 (13.5-16.2)	<0.0001	7.62 (6.89-8.43)	<0.0001
Metformin use										
No	4031	67.9	364516	79.0	368547	78.9	Ref.		Ref.	
Yes	1906	32.1	96637	21.0	98543	21.1	1.78 (1.69-1.88)	<0.0001	1.04 (0.96-1.12)	0.310

HCC, hepatocellular carcinoma; DM, diabetes mellitus NTD, New Taiwan Dollar; NAFLD, non-alcoholic fatty liver disease; ALD, alcohol-related liver diseases; HBV, hepatitis B virus; HCV, hepatitis C virus.
